# Multiple enhancers contribute to spatial but not temporal complexity in the expression of the proneural gene, *amos*

**DOI:** 10.1186/1471-213X-6-53

**Published:** 2006-11-09

**Authors:** Eimear E Holohan, Petra I zur Lage, Andrew P Jarman

**Affiliations:** 1 Centres for Integrative Physiology and Neuroscience Research, University of Edinburgh, George Square, Edinburgh EH8 9XD, UK; 2 Smurfit Institute of Genetics and TCIN, Lloyd Building, Trinity College Dublin, Dublin 2, Ireland

## Abstract

**Background:**

The regulation of proneural gene expression is an important aspect of neurogenesis. In the study of the *Drosophila *proneural genes, *scute *and *atonal*, several themes have emerged that contribute to our understanding of the mechanism of neurogenesis. First, spatial complexity in proneural expression results from regulation by arrays of enhancer elements. Secondly, regulation of proneural gene expression occurs in distinct temporal phases, which tend to be under the control of separate enhancers. Thirdly, the later phase of proneural expression often relies on positive autoregulation. The control of these phases and the transition between them appear to be central to the mechanism of neurogenesis. We present the first investigation of the regulation of the proneural gene, *amos*.

**Results:**

Amos protein expression has a complex pattern and shows temporally distinct phases, in common with previously characterised proneural genes. GFP reporter gene constructs were used to demonstrate that *amos *has an array of enhancer elements up- and downstream of the gene, which are required for different locations of *amos *expression. However, unlike other proneural genes, there is no evidence for separable enhancers for the different temporal phases of *amos *expression. Using mutant analysis and site-directed mutagenesis of potential Amos binding sites, we find no evidence for positive autoregulation as an important part of *amos *control during neurogenesis.

**Conclusion:**

For *amos*, as for other proneural genes, a complex expression pattern results from the sum of a number of simpler sub-patterns driven by specific enhancers. There is, however, no apparent separation of enhancers for distinct temporal phases of expression, and this correlates with a lack of positive autoregulation. For *scute *and *atonal*, both these features are thought to be important in the mechanism of neurogenesis. Despite similarities in function and expression between the *Drosophila *proneural genes, *amos *is regulated in a fundamentally different way from *scute *and *atonal*.

## Background

Proneural genes are key regulators of neurogenesis. They encode transcription factors of the basic-helix-loop-helix (bHLH) family whose expression endows ectodermal cells with competence to become neural precursors. For the *Drosophila *PNS, the proneural genes *achaete *(*ac*), *scute *(*sc*), *atonal *(*ato*) and *amos *are required for the precursors of different subsets of sense organs and sensory neurons (sense organ precursors, SOPs) [1-8]. Each is expressed in a complex ectodermal pattern that prefigures the formation of these subsets of SOPs. SOPs/sense organs are missing in loss of function mutants, whereas ectopic proneural gene expression results in induction of ectopic SOPs/sense organs.

Given their powerful effects in neurogenesis, it is not surprising that proneural genes are highly regulated. The study of their regulation illuminates the understanding of mechanisms of neurogenesis. For *ac *and *sc*, expression during SOP selection can be divided into two distinct phases [6-8]. Initially, the genes are expressed in undifferentiated ectoderm in a complex array of proneural clusters (PNCs) – groups of c.6–30 cells – in response to a 'prepattern' of upstream positional regulators. This initial expression pattern primarily determines the eventual locations of the sensory organs. Within each proneural cluster, *ac/sc *function triggers a process of mutual inhibition via activation of the Delta-Notch signalling pathway. In this process, each cell signals to inhibit *ac/sc *expression in adjacent cells of the cluster. A single cell (the SOP) eventually retains *ac/sc *expression at a high level, although the mechanism of this singling out is still not well understood [9]. However, it is clear that a critical aspect of the transition from PNC expression to SOP fate determination and subsequent neural development is the initiation of positive autoregulation [10,11]. Autoregulation allows high levels of *ac/sc *to accumulate in the SOP, which is probably a major factor in triggering the gene expression changes of neural development. Autoregulation is also important for *ato*. During R8 photoreceptor formation, Baker et al. [12] showed that *ato *expression evolves from an initial prepattern phase to an autoregulatory phase, and that Notch signalling directly inhibits the autoregulatory phase. These authors concluded that the transition from *ato*-independent prepattern regulation to autoregulation is critical for neural determination.

Analysis of proneural gene *cis*-regulatory elements illuminates these regulatory events. Genetic and reporter-gene evidence suggests that proneural cluster expression is driven by a modular battery of independently acting enhancers comprising much of the *ac-sc *complex. Each enhancer is thought to interact with a specific combination of prepattern transcription factors to drive proneural cluster expression in one or a few defined locations [13,14]. In the subsequent phase of SOP expression, regulation of the *sc *gene shifts to a single autoregulatory enhancer 3-kb upstream of the gene, called the SMC enhancer [11,15,16]. This element is thought to mediate the transition to SOP determination. Autoregulation is direct, via two E boxes that bind Sc/Daughterless heterodimers [11]. Other genes whose expression is triggered in SOPs also appear to have SOP enhancers of similar structure [15]. Thus a model has emerged in which proneural gene regulation can be divided into a early phase, which is dependent on upstream regulators, and a late phase, which is dependent on an autoregulatory enhancer [15].

The expression of *ato *is also regulated by a series of modular enhancers located in a region of about 15 kb surrounding the gene [17]. Like *sc*, it is suggested that *ato *has separate enhancers for the prepattern and autoregulatory phases of expression, with the former situated downstream of the gene and the latter upstream [17]. There appear, however, to be some important differences. The downstream region may comprise a single prepattern enhancer that drives *ato *expression in all its PNC locations [18]. This enhancer responds to *dpp *signalling and ecdysone. In contrast, subsequent SOP expression is driven by a modular array of enhancers – one for each location of *ato *expression. Furthermore, although the SOP enhancers have been postulated to be autoregulatory [17], direct autoregulation has only been shown for one of these, the '*ato *recruitment enhancer' [19].

In summary, a common theme in the regulation of *sc *and *ato *is the presence of separate enhancers for different temporal phases of expression. Where known, the enhancers for the second phase respond directly to autoregulation. A second theme is that spatial complexity in expression pattern represents the sum of the action of distinct enhancer modules. A major apparent difference between *sc *and *ato*, however, is that for *sc *the spatial complexity arises from multiple enhancers for the first temporal phase of expression, whereas for *ato *complexity seems to arise from multiple enhancers for the second phase.

In contrast to *ato *and *sc*, little is known of the regulation of *amos*. Here, we investigate whether these emerging themes of proneural gene regulation are shared by *amos *by characterising its expression pattern, and by identifying and characterising its *cis*-regulatory regions. The expression of *amos *has similarities and differences with *sc *and *ato *[2]. We show that Amos protein is expressed in a complex and dynamic pattern in the embryo that leads to the formation of precursors for a number of types of sense organ. The dynamics of *amos *expression resemble those of other proneural genes, with distinct temporal phases. Spatial complexity results from a series of site-specific enhancer modules that extend 3.5-kb upstream and 1-kb downstream from the transcription unit. Unlike *sc *and *ato*, however, there is no evidence for the existence of separate enhancers for different temporal phases of expression. In addition, we find no convincing evidence for autoregulation, thereby raising doubt about a general role for autoregulation in SOP formation.

## Results

### The dynamics of Amos protein expression during development

In order to analyse *amos *regulation, we characterised its expression pattern. Expression in the antennal imaginal discs has been described previously [1]. Here, expression begins at puparium formation in three distinct semicircles in the future third segment. By 8 h after puparium formation (APF), this expression merges into a single large crescent and continues until 16 hours APF. This expression is responsible for the third wave of olfactory SOP specification that takes place in the third antennal segment, which forms the precursors of the sensilla basiconica and trichodea [1,20].

**Figure 1:**
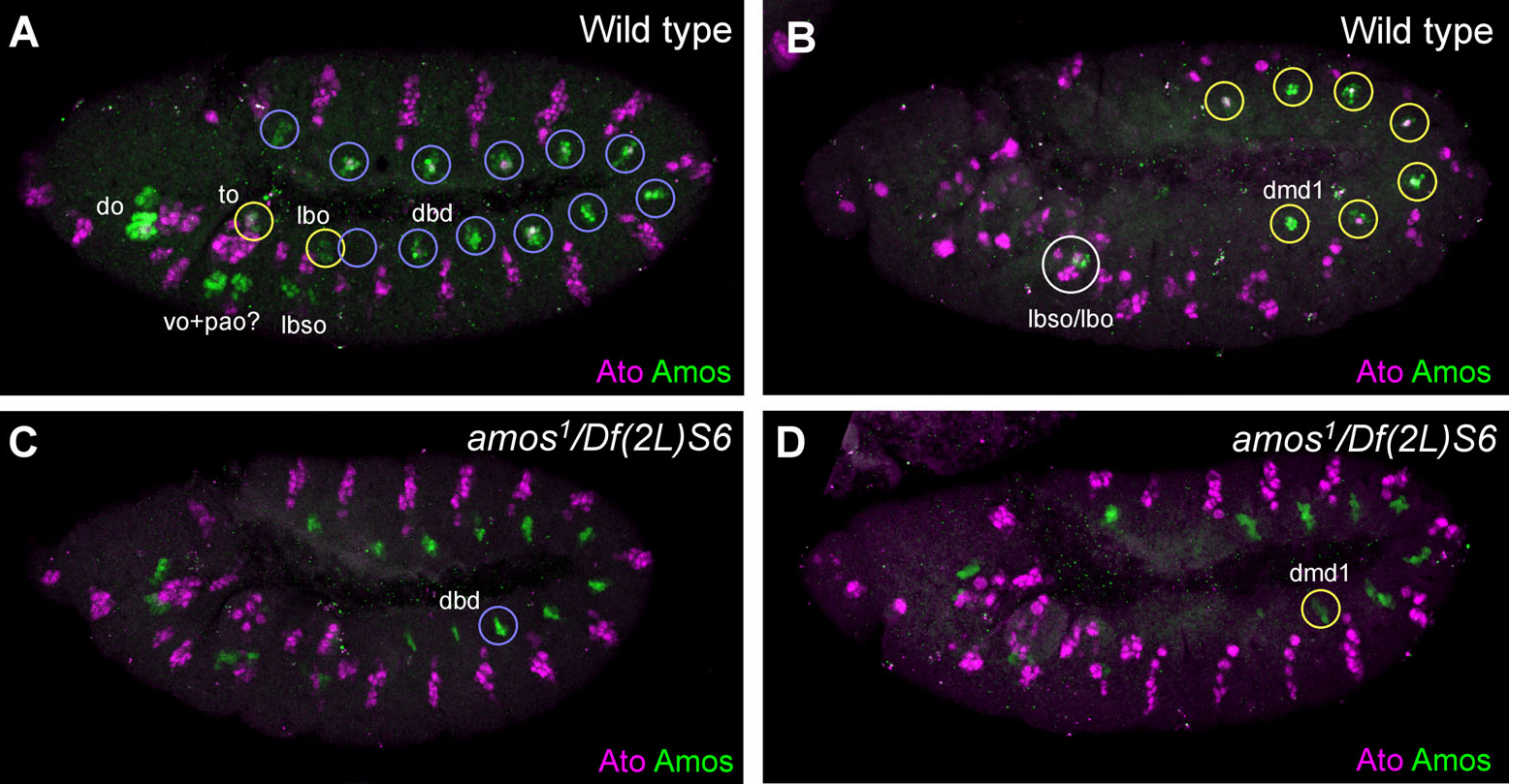
**Complex pattern and dynamics of Amos expression in the embryo**. Amos expression (green) relative to Ato expression (magenta) during neurogenesis. All embryos are lateral views unless otherwise stated. (A) Wild type, stage 10. Amos is expressed in PNCs for the **dbd **sensory neurons (blue circles – but no expression in segment T1) and a variety of head sense organs. Possible fates of the head organs are indicated. The **to **and **lbo **patches (yellow circles) appear homologous to the **dbd **PNCs. Ato is expressed in the SOP that arises from the **dbd **clusters. (B) Wild type, stage 11. Amos is now expressed in new clusters for the **dmd1 **neurons and **lbso **or **lbo **sense organ. Again, Ato is coexpressed with Amos in the trunk. (C) *amos*^*1 *^mutant embryo, stage 10. Ato expression in the **dbd **cells is abolished. (D) *amos*^*1 *^mutant embryo, stage 11. Ato expression in the **dmd1 **cells is abolished. Note that truncated Amos protein is detected in the *amos*^*1 *^embryos [1]. **do **= dorsal organ; **to **= terminal organ; **vo **= ventral organ; **pao **= papilla organ; **lbo **= labial organ; **lbso **= labial sense organ; **dmd **= dorsal multiple dendritic neuron; **dbd **= dorsal bipolar neuron.

In the embryo, *amos *RNA is expressed transiently in a segmentally repeated pattern of presumed proneural clusters and SOPs at stages 10/11 [2,21]. In the trunk, Amos protein expression begins at stage 10 in single small cluster in the dorsal ectoderm of each abdominal segment and of thoracic segments T2 and T3 (Fig. 1A). This cluster is absent from T1. Shortly afterwards, Amos expression ceases here. At this time, in abdominal segments A1–A7 only, transient expression begins in a second small cluster of cells adjacent and ventral to the first cluster (Fig. 1B). Subsequent analysis (below) shows that the first clusters give rise to the **dbd **neurons and the second clusters to the **dmd1 **neurons. Interestingly, the presumed SOPs that derive from both these clusters (but not the clusters) also expresses the related proneural protein, Atonal (Fig. 1A,B). The expression of Ato in these SOPs requires *amos *but the converse is not true, confirming that Amos provides the proneural function for these SOPs (Fig. 1C,D and data not shown).

In the stage 10 embryonic head, Amos is expressed in large ectodermal clusters in the antennal, maxillary, and labial segments (Fig. 1A). These are rather more ventral than the clusters in the trunk. Slightly later, expression appears in small clusters in the maxillary, mandibular and labial primordia. Expression also appears in small clusters that appear homologous to those in the trunk. The head expression suggests that *amos *may function in the formation of a variety of head sense organs.

### Identification of sequences required for *amos *transcription

**Figure 2:**
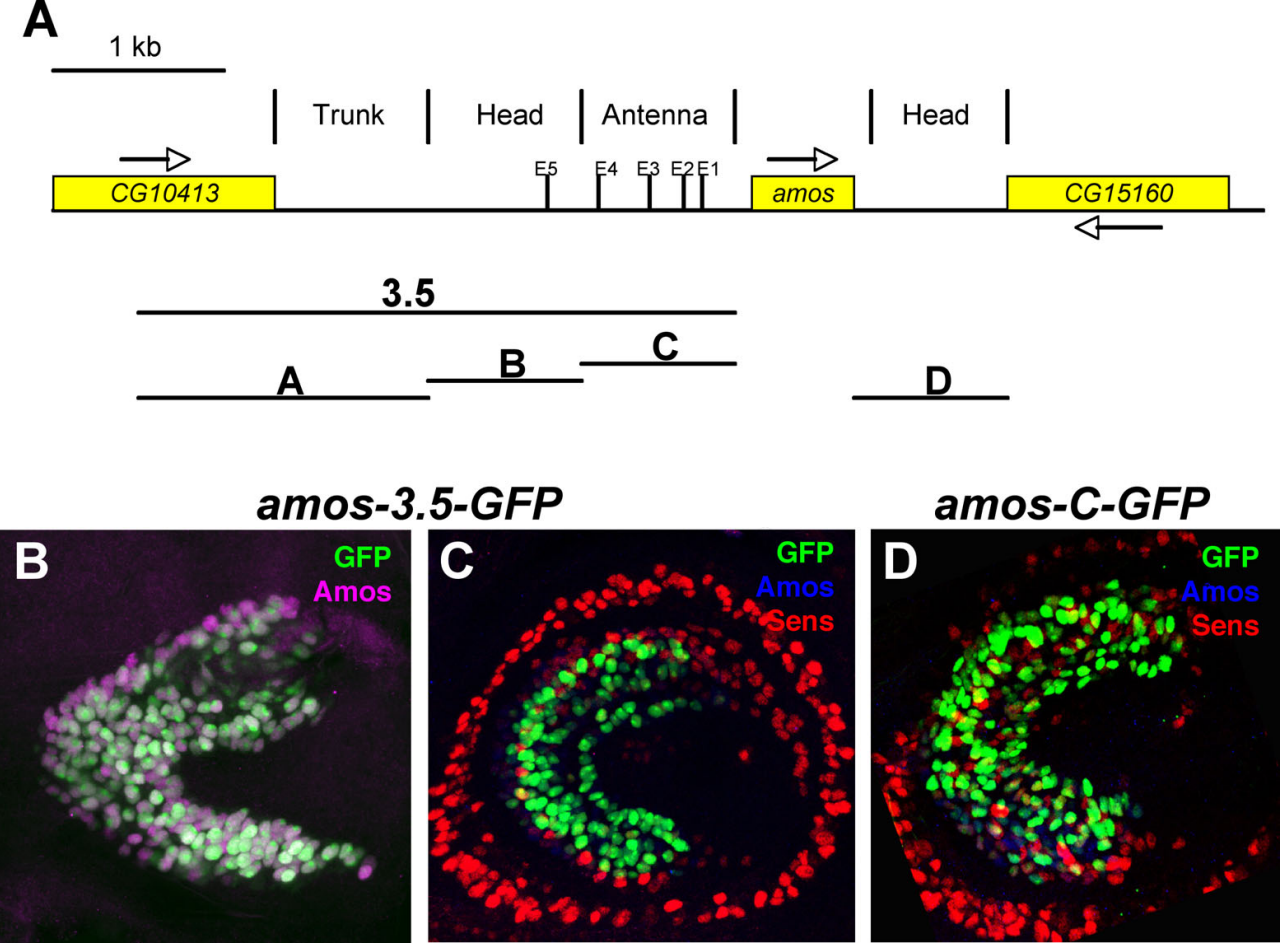
**Enhancer elements flanking the *amos *gene**. (A) Schematic of the *amos *genomic location summarising the locations of nearest neighbouring genes, fragments tested for enhancer activity, location of enhancer activity, and location of potential proneural protein-binding E box sites. (B–D) Antennal imaginal discs, 8 h after puparium formation. (B) *amos-3.5-GFP*, GFP (green) is almost completely coexpressed with Amos (magenta). stained to detect GFP (green), Amos (blue) and Sens (red) as an SOP marker. (C, D) GFP (green) is coexpressed in some cells with Amos (blue) and Sens (red) in *amos-3.5-GFP *(C) and *amos-C-GFP *(D). Expression of *amos-C-GFP *is lower than that of *amos-3.5-GFP*, although this is not apparent from these images.

**Figure 3:**
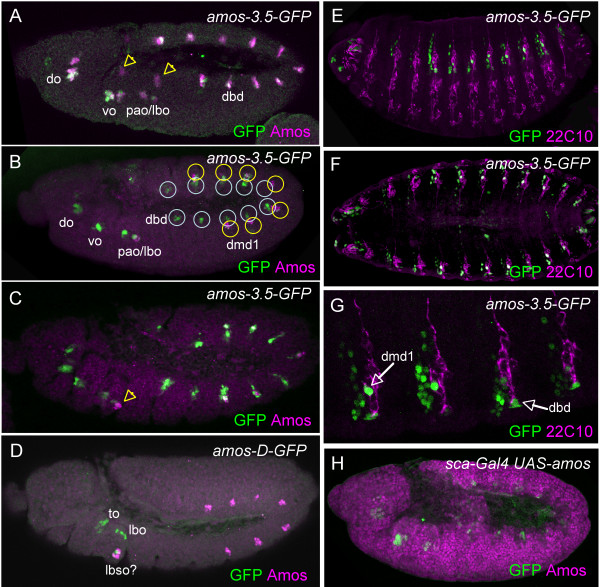
**Regions flanking the *amos *gene support GFP expression in all embryonic locations in which *amos *is expressed**. (A–C) *amos-3.5-GFP *in early embryos, GFP expression (green) relative to Amos (magenta). (A) Stage 10. Most areas of Amos expression overlap with GFP, but arrows indicate two areas of Amos expression that are not mimicked by this reporter gene construct. (B) Stage 11. **dbd **(blue) and **dmd1 **(yellow) clusters are circled. (C) Stage 12. Arrow indicates a third region with no overlap of expression. (D) *amos-D-GFP*, stage 10. GFP is present in Amos-expressing clusters that correspond to the three areas arrowed in A and C. (E–G) *amos-3.5-GFP *in stage 15 embryos showing perdurance of GFP (green) relative to the sensory neuron marker, 22C10 (magenta). **dmd1 **and **dbd **neurons are arrowed in G. (H) *amos-3.5-GFP *in *sca-Gal4 UAS-amos *embryo. Widespread misexpression of Amos (magenta) does not result in ectopic expression of GFP (green) from this reporter construct. Abbreviations as in Fig. 1.

To identify the *cis*-regulatory sequences of *amos*, the intergenic regions upstream and downstream were tested for their ability to drive expression of a GFP reporter gene (Figs 2,3, and Table 1). A construct with a 3.5-kb upstream fragment (*amos-3.5-GFP*) supports GFP expression in the third antennal segment. The expression pattern was characterised by co-labelling with antibodies to Amos and Senseless (Sens) as a marker of SOPs [22]. Over a period of 0–8 h APF, GFP expression was largely coincident with Amos protein (Fig. 2B,C), suggesting that the fragment contains a major enhancer for expression of Amos in the antennal disc. Whilst generally co-expressed, GFP is not observed strongly in the cells in which Amos is most recently activated. This appears to represent a slower induction of GFP synthesis and maturation relative to endogenous proneural proteins [23]. A subset of GFP-expressing cells (with deeper nuclei) also express Sens, thus representing the *amos*-dependent olfactory SOPs themselves (Fig. 2C). GFP-negative SOPs are also present, which are likely to represent the earlier waves of *ato*-dependent olfactory SOPs. Thus, *amos-3.5-GFP *contains a major enhancer that drives Amos expression in most or all cells of the olfactory PNCs. Strong expression in the resulting SOPs suggests that the fragment drives expression in both the PNCs and SOPs, although it is also possible that GFP expression in SOPs represents perdurance of expression driven by the PNC enhancer. Previously, the enhancer activity of a 56-bp larger fragment had been tested by cloning into a Gal4 expression vector [1]. The expression pattern of *amos-3.5-GFP *described here differs from that described for *amos-3.6-Gal4 *in that the latter appeared largely SOP-specific [1]. It is possible that this might represent a strongly delayed onset of GFP expression from this construct.

**Table 1:** Summary of GFP expression locations observed when driven by *amos *flanking fragments

	GFP reporter expression			
Fragment	Head	Trunk	Antenna	Ectopic

3.5	do, vo, lbo	dmd, dbd	+	–
A	–	dmd, dbd	–	–
B	do, vo, lbo	–	–	+ (trunk)
C	–	–	+	+ (trunk, ant.)
D	to, lbso	–	–	–

*amos-3.5-GFP *also supports expression in the embryo. The pattern of GFP closely resembles that of Amos protein although the appearance of GFP is delayed (Fig. 3A–C). Owing to the transient nature of Amos expression, this means that relatively little overlap of Amos and GFP expression are observed. Between stages 10 and 11, GFP expression is detected in the same sequence of cell clusters as Amos in the head and trunk. Expression begins in the head antennal and maxillary segments. It is then observed in the other head clusters and the first thoracic and abdominal clusters. In the latter, GFP expression appears in the first clusters as Amos expression disappears from them and is replaced by expression in the second more ventral cluster in A1–7 (Fig. 3B,C).

By stage 12, *amos *expression has been turned off. However, perdurance of GFP expression was used to follow the fate of the different *amos *clusters. There is a complex network of sensory neurons in the trunk of the embryo, but *amos *is responsible for only two of the multidendritic neurons, the **dbd **and **dmd1 **neurons [1,21]. In the abdominal segments of late embryos, *amos-3.5-GFP *expression is observed strongly in the dorsally located dmd1 neuron in abdominal segments A1–7 (as marked by the sensory neuron marker 22C10 (anti-Futsch)) (Fig. 3E–G). Weaker and variable expression is observed in the dbd neuron and its associated glial cell in segments T2,3 and A1–8/9. In addition, some ectodermal cells also express GFP, which is consistent with perdurance in some of the PNC cells. GFP is not expressed in neurons in T1, which is consistent with observation that *amos *is not expressed in this segment. Interestingly, the lack of expression in T1 suggests that this segment does not possess a **dbd **neuron. Similarly, only segments A1–7 appear to have a GFP-expressing **dmd1 **neuron. Consistent with this, a marker of the **dbd **and **dmd1 **cells (anti-Pdm) detects no neurons in T1, and only the **dbd **neuron in T2,3 (data not shown). Interestingly, the dorsoventral locations of the **dbd **and **dmd1 **neurons appear reversed relative to their proneural clusters, suggesting that one or both neurons undergo migration (cf Fig. 3B and 3G).

GFP expression also perdures in the head region. This is particularly associated with the complex clusters of sense organs that form the antennomaxillary complex and pharynx-associated sense organs of the larva. Perdurance of expression confirms that in the antennal segment *amos *contributes to the larval olfactory organ, the dorsal organ (**do**) (see [24] for nomenclature). The main expression in the maxillary segment contributes to the ventral organ (**vo**), and perhaps the papilla organ (**pao**), whose neurons are reported to resemble **dbd **neurons [24]. Equivalent expression in the labial segment contributes to the labial sense organ (**lbso**).

*amos-3.5-GFP *is expressed in all locations of Amos expression except for three small areas dorsally in the maxillary and labial segments (arrowed in Fig. 3A,C). To locate the enhancer sequences responsible for these areas, a 1-kb region downstream of *amos *was also tested for enhancer activity (construct *amos-D-GFP*; Fig. 2A). This region supports GFP expression in these three groups of cells (Fig. 3D). Their location and GFP perdurance suggests that these appear to contribute neurons of the terminal organ (**to**), labial organ (**lbo**), and labial sense organ (**lbso**). Between them, the upstream and downstream flanking regions contain enhancers that can account for the entire *amos *expression pattern.

### The *amos 3.5-kb *region consists of separable enhancer modules

**Figure 4:**
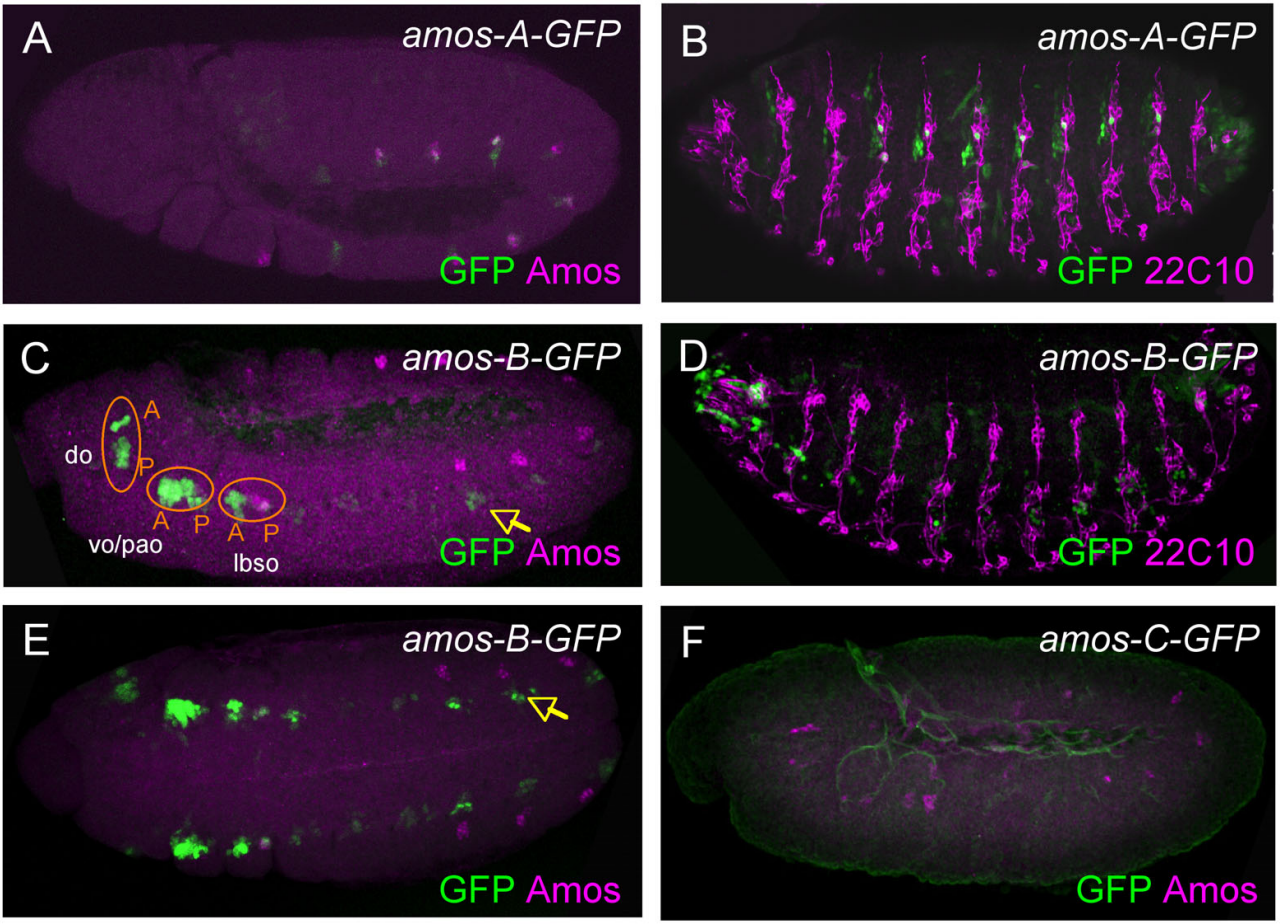
**The *amos *upstream flanking region contains multiple enhancers**. (A, C, E, F) Stage 10/11 embryos showing GFP (green) and Amos (magenta). (B, D) Stage 15 embryos showing GFP (green) and 22C10 (magenta). (A, B) *amos-A-GFP *supports expression in the trunk. (C, D, E) *amos-B-GFP *supports expression in the head, and also shows ectopic expression in the trunk. (E) is a ventral view. (F) *amos-C-GFP *does not support expression in the embryo.

The complex nature of *amos *expression suggests that the 3.5-kb fragment may contain different enhancers for different aspects of pattern. The 3.5-kb fragment was subdivided into three smaller fragments (A, B, C) measuring 1.68 kb, 961 bp and 893 bp. *amos-A-GFP *is expressed solely in the trunk of the embryo: there is no GFP expression in either the antennal disc or the head region of the embryo (Fig. 4A and data not shown). This suggests that an enhancer element responsible for *amos *expression in the **dbd **and **dmd **clusters is present in fragment A. In late embryos, perduring GFP was seen in the trunk in a similar pattern to that of *amos-3.5-GFP *(Fig. 4B).

*amos-B-GFP *also supported expression in the embryo but not the antennal disc. In this case, GFP was observed in the head region in a pattern similar to that supported by *amos-3.5-GFP *(Fig. 4C). Thus, an enhancer(s) for *amos *expression in the head is present in fragment B, and this appears to be required for most of the components of the *amos *head pattern, as confirmed by 22C10 staining of late embryos (Fig. 4D). *amos-B-GFP *is also expressed in a pattern of ectodermal clusters in the trunk, but this expression appears to be ectopic: the clusters are more ventral than those for *amos-3.5-GFP *or for Amos itself, and the expression does not perdure into cells associated with the PNS (Fig. 4C,D). Interestingly, this ectopic expression appears to resemble in its segmental location the *amos-B-GFP *pattern observed in the head (Fig. 4E). This suggests that the ectopic trunk pattern represents the inappropriate activity of the head enhancer present in B. In contrast, a construct combining fragments A and B (*amos*-AB-GFP) shows correct head and trunk expression (data not shown). There may therefore be an inhibitory sequence in A that normally restrictsthe activity of the B enhancer to the head.

*amos-C-GFP *does not support expression in the embryo at the time that *amos *is normally expressed (Fig. 4F). However, as the neurons start to differentiate, GFP expression is switched on in an inconsistent subset of cells marked by 22C10 (data not shown). This presumably represents artefactual expression. In contrast, fragment C drives expression in the antennal imaginal disc (Fig. 2D). GFP expression is present in the *amos *dependent SOPs and in some cells of the proneural cluster. There is no discernible difference between the location of GFP expression as driven by the *amos-3.5-GFP *fragment and that driven by *amos-C-GFP*, although expression from the latter is generally weaker.

In summary, distinct enhancer sequences are required for *amos *expression in the embryo and antennal disc. More than one enhancer is responsible for *amos *expression in the embryo, and head and trunk enhancers appear to be separate. The presence of enhancer modules for different expression locations is consistent with the findings of other proneural genes. However, our analysis found only one enhancer for each location of *amos *expression.

### A single enhancer can rescue of olfactory sensillum loss in *amos *mutants

For *sc *and *ato*, experiments investigating transgene rescue of sensillum loss in mutants showed that substantial phenotypic rescue is achieved if a transgene includes enhancers for both phases of expression [11,17,25]. In contrast, a transgene driven by a single enhancer (for either the first or second phase of expression) rescues poorly. In the case of *amos*, we have found no separation of enhancers for temporal phases. Although distinct phases of *amos *expression can be discerned, they appear to be driven by a single element in each location. It seems unlikely that further subdivision will reveal such separable enhancers, nor that other enhancers exist farther up- or downstream of *amos*. We therefore determined whether the 3.5-kb region contained all elements responsible for *amos *regulation in the antennal disc in a rescue experiment. The *amos*-3.6-Gal4 line was used to drive UAS-*amos *expression in *amos *mutant flies (*amos*^*1*^*/Df(2L)M36-S6*), and antennae from such flies were examined for types and numbers of sensilla on the third segment. Mutation of *amos *results in the loss of all sensilla trichodea and sensilla basiconica, as well as the appearance of ectopic sensory bristles [1]. Expression of *amos *driven by the *amos*-3.6-Gal4 line resulted in a substantial rescue of this defect. Ectopic bristles were almost completely suppressed (average of 1.66, cf c.20 for *amos*^*1*^*/Df(2L)M36-S6*). Sensilla trichodea were present in numbers close to that expected in wildtype (66.7 ± 11.0). Substantial numbers of sensilla basiconica were also present, although less than half the number expected for wildtype (84.0 ± 10.0). Although quantitatively not complete, the degree of rescue suggests that all major patterning elements necessary for *amos *expression in the antenna are present in *amos*-3.6-Gal4 and also, by inference, in the 3.5-kb fragment. Lack of complete rescue may reflect the delay in onset of Gal4-driven expression in this system. Interestingly, the numbers of *ato*-dependent sensilla coeloconica are reduced compared to wildtype (34.7 ± 4.2). Such reduction is also seen when *amos *is misexpressed in the wildtype antenna (S. Maung and APJ, in prep.). It is possible that perdurance of Gal4-driven expression of *amos *interferes with endogenous *ato *function.

### Regulation of enhancer elements: is there an autoregulatory component?

Where known for other proneural genes, the second phase of expression involves direct autoregulation, with proneural/Daughterless protein heterodimers binding to E box sequences within an autoregulatory enhancer. We asked whether autoregulation is also important in *amos *regulation, concentrating on the antennal disc expression. If *amos *is autoregulatory, one might expect functional E box binding sites to be present within the *amos-C *antennal regulatory region. Four potential E box sequences (CANNTG) are present in this fragment, two of which are conserved between *Drosophila melanogaster *and *D. pseudoobscura *(data not shown). None of these sequences (ttCAAGTGa, aaCAATTGt, gtCATATGg, gtCATTTGg) conform completely to the consensus sequences reported for Sc (gCAG(G/C)TG(g/t)) or Ato (a(a/t)CA(G/T)GTG(g/t) (Singson et al., 1994; Powell et al., 2004). However, although Amos protein is predicted to function via E box binding, no such site has yet been characterised. Therefore, we investigated whether any of these E box sequences are important for *amos-C *enhancer function by mutating all four E boxes within the *amos-C-GFP *construct (*amos-Cmut-GFP*). However, no clear reduction in expression was observed for *amos-Cmut-GFP *compared with the unmutated construct (data not shown). In case autoregulation lies outside fragment C, the whole of the 3.5-kb sequence was scanned for E box sequences. No further E boxes matching the known consensus sequences for Sc or Ato were found. The closest match is a site of atCAGGTGa (differing from the Ato consensus sequence in its 3' flanking base). This sequence is conserved in *D. pseudoobscura*. However, when mutated within *amos*-3.5-GFP, no difference in GFP expression pattern was observed in the antenna or embryo (data not shown).

Autoregulation may occur indirectly via the regulation of an intermediate factor. To find evidence for indirect autoregulation, we determined whether misexpression of *amos *results in ectopic induction of *amos-3.5-GFP*. In the embryo, no ectopic expression was observed from *amos-3.5-GFP *when UAS-*amos *was driven in the ectoderm by a *sca*-Gal4 driver (Fig. 3H). Using the *Gal4*^109-68 ^line in imaginal discs in third instar larvae [26], no ectopic *amos-3.5-GFP *expression was observed upon *amos *misexpression, except for a small number of GFP-expressing cells in antennal discs. However, variable numbers of these cells were also visible in control antennal discs that lacked the UAS-*amos*, and so appear to represent a genetic background effect (data not shown).

*amos-3.5-GFP *expression was also examined in *amos *mutant embryos to look for loss of GFP expression that might indicate the need for autoregulation. In such embryos, no clear difference from wild type was observed in the GFP expression pattern (data not shown). In mutant antennal discs, ectodermal GFP expression appeared unchanged, although SOP expression was lost as would be expected from the absence of such cells (data not shown). In summary, no part of the *amos *expression pattern could clearly be seen to depend on endogenous *amos *expression.

## Discussion

The proneural gene, *amos*, is expressed during the development of a variety of sensory structures. In the embryonic trunk it is only expressed during formation of the **dmd1 **and **dbd **neurons. In the head, however, *amos *has the potential to influence the development of many cephalic sense organs, the functions of which are poorly known. Given the role of *amos *in adult PNS development, its expression in the embryonic head marks candidate olfactory organs. There is evidence, however, that larval olfaction is carried out by the dorsal organ [27], and so the role of *amos *in other head sense organs remains to be determined. In some instances, *amos *appears to activate *ato *expression in sensory precursors. This raises the possibility that the combination of the two factors may play a distinctive role in neuronal subtype specification.

### Modular arrangement of *amos cis*-regulatory regions gives rise to complex spatial regulation

Functional dissection of the regions around the *amos *gene has shown that it is regulated via an array of separable enhancers, both upstream and downstream. Thus, a complex expression pattern results from the sum of a number of simpler sub-patterns driven by specific enhancers. In this respect, *amos *conforms well to the regulation characteristics of other *Drosophila *proneural genes. Vertebrate proneural gene homologues also have modular enhancer arrangements [28,29]. One notable feature is that the separation of head and trunk enhancers appears to be unique to *amos*.

At least in the case of *ac/sc*, individual modules respond to different combinations of upstream (prepattern) factors [13,30]. For *amos*, one likely prepattern factor is the runt domain transcription factor, Lozenge, whose function is required for *amos *expression in the antennal disc [2]. The presence of three potential Lozenge binding sites in the *amos *fragment C suggests that *amos *may be under the direct control of Lozenge (unpublished observations).

### No separable enhancers for distinct temporal phases of regulation

Investigation of *sc *regulation has led to a paradigm in which it is regulated in two phases via different enhancers. In the first PNC phase, *sc *is regulated via enhancers that respond to upstream prepattern factors. In the SOP phase, it is regulated via a single SOP enhancer, with positive autoregulation as a major input [15]. For *ato*, there is also evidence for two separable regulatory phases, although separation is less clear. For instance, an autoregulatory enhancer has been described, but this only functions in the specific context of SOP recruitment via EGFR signalling [19]. Furthermore, embryonic expression has been described as being initiated by a 3' 'prepattern' enhancer, and then maintained by a 5' enhancer with an autoregulatory component [17]. However, the 5' enhancer is not SOP-specific, and there is no direct evidence that it responds to autoregulation (S. Cachero and APJ, unpublished observations). Nevertheless, whilst differing in important details from the *sc *paradigm, it is clear that *ato *is regulated in different temporal phases via distinct enhancers [17].

Given these observations, it is surprising that we found no evidence that *amos *is regulated in distinct temporal phases via separate enhancers. Although it is possible that the enhancers identified here could be subdivided further, there is currently no obvious separation of PNC and SOP enhancers. This suggests either that enhancers for separate phases of expression are not fundamental to proneural gene function in neurogenesis, or that *amos *regulation and function differs substantially from that of other proneural genes.

### Autoregulation during proneural gene function

For *sc *regulation, the rationale for separate temporal enhancers is that the mechanism of neurogenesis depends on a progression from an initial competence phase in the PNC to a commitment phase in the SOP. Separable enhancers allow the first phase to be achieved by an array of prepattern enhancers and the second phase by a positive autoregulatory enhancer. The latter contains two E-box binding sites responsible for the autoregulation [10,11]. A similar enhancer seems to exist for *ac*: a 0.9 kb enhancer upstream of *ac *has three E-boxes that are important for its function [10,31,32].

For *amos*, we found no evidence for autoregulation. From the close similarity of their bHLH sequences, it is expected that Amos and Ato proteins would have similar DNA binding characteristics. However, no E box could be identified as being required for *amos *enhancer function. It is possible that autoregulation is indirect, or occurs directly via a protein-protein interaction with another DNA binding cofactor. However, no ectopic enhancer activity could be observed after *amos *misexpression. Whilst contrary to the regulatory trends established for *sc *and *ato*, the lack of evidence for *amos *autoregulation nonetheless correlates with the lack of separable temporal enhancers.

The lack of *amos *autoregulation is surprising. The autoregulatory elements of *sc *and *ato *are the target of other regulatory inputs, so that modulation of autoregulation (facilitation or inhibition) during the PNC to SOP transition is an important factor in limiting SOP determination. The *sc *SOP enhancer is thought to be a direct target of E(spl) proteins and other factors during lateral inhibition [11]. The *ato *recruitment enhancer has a binding site for Pointed protein next to the functional E box, which is crucial for allowing autoregulation [19]. This ensures that autoregulation (and SOP commitment) is triggered when a competent cell (in the PNC) receives EGFR signalling input. For *sc *and *ato*, modulation of autoregulation allows precise control neurogenesis. Lack of *amos *autoregulation may reflect the fact that *amos *function results in large numbers of SOPs, at least in the antenna, and so such precise limitation on its activity may not be necessary. Interestingly *amos *also appears to be a more powerful proneural gene in other assays (S. Maung and APJ, in prep)[33,34]. Another intriguing possibility is that the need for proneural autoregulation correlates not with SOP commitment, but instead with the need to maintain expression in the SOP in situations where SOP expression is relatively prolonged. At least in the embryo, Amos expression in SOPs indeed appears to be very transient (and hence the limited overlap with GFP in reporter gene lines); such transient expression may not require an autoregulatory input.

Our findings suggest that autoregulation is not a universal feature linking proneural gene function to neurogenesis. For *ato*, only in the case of one enhancer has direct positive autoregulation been demonstrated so far [19]. For vertebrate proneural homologues, direct autoregulation via a conserved E box has been demonstrated for Math1 [35]. On the other hand, there is no evidence of positive autoregulation for Mash1 [36] or neurogenin [37]. It is far from clear how general the occurrence of autoregulation is in proneural gene function.

## Conclusion

Unlike *sc *and *ato*, the proneural gene, *amos*, does not seem to depend on autoregulation, via separable enhancers, to promote SOP determination. The mechanisms proposed for the role of autoregulation and distinct temporal enhancers in neurogenesis must be modified.

## Methods

### Fly stocks

Fly stocks used were *amos*^*1 *^[1], *Df(2L)S6 *[1], UAS-*amos *[2], UAS-*ato *[5], *109-68Gal4 *[26] and *scabrous-Gal4*.

### Reporter plasmid constructs

Primers were designed to amplify a 3.5-kb fragment upstream of the *amos *gene (5'-GGAGTGCAACCGGATTTAACC and 5'-CCCGATGCCAACCTCTTGA). Three further amplifications subdivided this fragment into three sections: *amos-A *(1.68 kb) (5'-GGAGTGCAACCGGATTTAACC and 5'-CCTAGCGAAAGCGGAGAATT), *amos-B *(961 bp) (5'-AATTCTCCGCTTTCGCTAGG and 5'-CGAGGAGTTCGCTGAATTTC), and *amos-C *(893 bp) (5'-GAAATTCAGCCAACTCCTCG and 5'-CCCGATGCCAACCTCTTGA). Primers were also designed to amplify a 1-kb fragment downstream of the *amos *gene (*amos-D*) (5'-GTATGAAATGGTGGAGTTGG and 5'-CGAACTCAGGTGTCTTTAGA). All five fragments were cloned into pHStinger [38] to give *amos-3.5 GFP, amos-A-GFP, amos-B-GFP, amos-C-GFP *and *amos-D-GFP *for germ line transformation.

### Site directed mutagenesis

E boxes were mutated using the Stratagene Quikchange mutagenesis kit. In each case the sequence was changed to GGATCC.

### *Drosophila *germ line transformation

Transformation plasmids were injected into *w^1118^*; Δ*2–3 *flies. Transformants were selected and outcrossed to *w^1118 ^*to remove the Δ*2–3 *element. At least two independent lines were analysed for each construct.

### Immunohistochemistry

Antibody staining of pupal antennae was carried out as previously described [2]. Pupae were staged by collecting at the time of puparium formation and then aging on moist filter paper at 25° before dissection. For misexpression analysis, imaginal discs were dissected from wandering third instar larvae and fixed in 3.7% formaldehyde (10 min at room temperature). Embryos were collected, fixed and stained according to standard procedures. Incubations with primary and secondary antibodies were carried out according to standard procedures. Primary antibodies used were mouse anti-22C10 (1:200; obtained from the Developmental Biology Hybridoma Bank, Iowa City, Iowa), mouse and rabbit anti-GFP (1:500; Molecular Probes), guinea-pig anti-Sens (1:6250; [22], and rabbit anti-Amos 1:1000 [1]). Secondary antibodies (1:1000) were obtained from Molecular Probes. Confocal microscopy analysis was carried out using a Leica TCS SP2 or a Zeiss PASCAL microscope.

## Authors' contributions

EEH designed and carried out most of the experiments and helped draft the manuscript. PIzL scored antennal olfactory sensilla and carried out pupal antennal dissection and immunohistochemistry. APJ conceived the study, participated in its design and coordination, carried out the *amos/ato *expression analysis, and drafted the manuscript.
